# Optimal reconstruction methods after distal gastrectomy for gastric cancer: A systematic review and network meta-analysis

**DOI:** 10.1097/MD.0000000000010823

**Published:** 2018-05-18

**Authors:** Zhaolun Cai, Ye Zhou, Chenxiao Wang, Yiqiong Yin, Yuan Yin, Chaoyong Shen, Xiaonan Yin, Zhixin Chen, Bo Zhang

**Affiliations:** a Department of Gastrointestinal Surgery, West China Hospital, Sichuan University, Chengdu, Sichuan; b Department of Gastric Surgery, Fudan University Shanghai Cancer Center, Shanghai; c West China School of Preclinical Medicine and Forensic Medicine, Sichuan University, Chengdu, Sichuan, China.

**Keywords:** Billroth I, Billroth II, gastric cancer, network meta-analysis, reconstruction, Roux-en-Y

## Abstract

**Background:**

The choice of anastomosis methods including Billroth I, Billroth II, and Roux-en-Y after a distal gastrectomy is still controversial. The conventional meta-analyses assessing 2 alternative treatments were not powered to compare differences in clinical outcomes. To guide treatment decisions in patients with gastric cancer (GC) after distal gastrectomy, we did a systematic review and network meta-analysis to identify the best reconstruction method.

**Methods:**

We systematically searched PubMed, EMBASE, the Cochrane Library for randomized controlled trials comparing the outcomes of Billroth I, Billroth II, or Roux-en-Y reconstruction after distal subtotal gastrectomy for patients with GC, then we performed a direct meta-analysis and Bayesian network meta-analysis to pooled odds ratios (OR) or weighted mean differences (WMD) with 95% credible intervals (CrI) with random effects model. The node-splitting method was used to assess the inconsistency. We estimated the potential ranking probability of treatments by calculating the surface under the cumulative ranking curve for each intervention.

**Results:**

Nine studies involving 1161 patient were included in the network meta-analysis. Statistical significance was reached for the comparisons of Roux-en-Y versus Billroth I reconstruction (WMD 37, 95% Crl: 22–51) and Billroth II versus Billroth I reconstruction (WMD 25, 95% Crl: 5.8–43) for operation time; and Roux-en-Y versus Billroth I reconstruction (WMD 26, 95% Crl: 2.1–68) for intraoperative blood loss; and Roux-en-Y versus Billroth I reconstruction (OR 3.4, 95% Crl: 1.1–13) for delayed gastric emptying. Roux-en-Y reconstruction was superior to Billroth I and Billroth II reconstruction in terms of frequency of bile reflux (OR 0.095, 95% Crl: 0.010–0.63; OR 0.064, 95% Crl: 0.0037–0.84, respectively) and the incidence of remnant gastritis (OR 0.33, 95% Crl: 0.16–0.58; OR 0.40, 95% Crl: 0.17–0.92, respectively).

**Conclusion:**

Roux-en-Y reconstruction is superior to Billroth I and Billroth II reconstruction in terms of preventing bile reflux and remnant gastritis, Billroth I and Billroth II anastomosis could be considered as the substitute in consideration of technical simplicity. As for postoperative morbidity and the advantage of physiological food passage, Billroth I method is the choice.

## 1 Introduction

Gastric cancer (GC) is the third most common cause of death from cancer worldwide, accounting for 6.8% of the total cases and 8.8% of total deaths with mortality number of 723,000 in 2012.^[1]^ Complete surgical resection is the only curative therapeutic option for patients with localized GC.^[2]^ For most GC the in the lower two-thirds of the stomach, distal gastrectomy is the recommended surgery, however, the choice of anastomosis method after a distal gastrectomy is still controversial. Various reconstruction methods have been introduced to improve perioperative care and to reduce postoperative complications since Billroth conducted the first subtotal gastrectomy in 1881.^[3]^ Billroth I (B-I), Billroth II (B-II), and Roux-en-Y (R-Y) are all valid reconstruction methods. B-I and B-II reconstructions are preferred in Asia for their procedure simplicity. However many patients have obvious complications after surgery including gastroesophageal and reflux symptoms.^[4]^ R-Y gastrojejunostomy is more commonly performed in Western countries with an attempt to prevent alkaline reflux gastritis and reflux esophagitis.^[5]^ Despite its advantages, patients undergoing R-Y reconstruction often experience delayed gastric emptying, nausea, vomiting, or abdominal pain, making surgeons reluctant to conduct the procedure.^[6,7]^ Thus, there is still controversy regarding which is the best reconstruction method.

The conventional meta-analyses assessing 2 alternative treatments were not powered to derive comparative evidence when there were no head-to-head comparisons. Thus we performed a direct meta-analysis and Bayesian network meta-analyses to investigate the question, combining direct and indirect comparisons to derive comparative evidence ^[8–11]^ of B-I, B-II, and R-Y reconstruction for patients with GC after distal gastrectomy.

## 2 Method

### 2.1 Search strategy

Two investigators performed a systematic literature search in PubMed, EMBASE (Ovid), Cochrane Library (Ovid), (last updated on December 5, 2017) without language restriction, using combinations of the following terms: “stomach neoplasms,” “gastric cancer,” “stomach cancer,” “Billroth I,” “Roux-en-Y,” “Billroth II,” “reconstruction,” “anastomose,” “randomized controlled trial,” “controlled clinical trial,” “random allocation,” “double-blind method,” “single-blind method,” “survival analysis,” “treatment outcome” in accordance with the Cochrane Handbook for Systematic Reviews of Interventions.^[12]^

The reference list was also checked for relevant studies, and all studies were carefully evaluated to identify duplicate data.

### 2.2 Study selection

The following criteria were used for the study selection: Participants (P): Patients were eligible if they had histologically proven gastric located in the antrum, angle or lower body of the stomach; no evidence of distant metastasis; interventions (I) and comparisons (C): Studies that compared B-I, B-II, or R-Y reconstruction after distal gastrectomy for GC were included in this meta-analysis. Outcomes: Surgical characteristics, early postoperative outcomes and the results of the postoperative endoscopic examination were evaluated. Operation time, intraoperative blood loss, and hospital stay were the main surgical characteristics to be assessed. Early postoperative outcomes included anastomotic leakage and stricture and delayed gastric emptying. The postoperative endoscopic examination includes bile reflux, food residual, remnant gastritis, and reflux esophagitis. Study design (S): Published randomized controlled trials (RCTs); Provided enough information to surgical characteristics, early postoperative outcomes and the results of postoperative endoscopic examination.

Conference abstracts, letters, case reports, reviews, studies without randomization for treatment allocation or studies without usable data were excluded.

### 2.3 Assessment of risk of bias and data collection

Qualitative assessment and data extraction were finished by 2 investigators independently, and disagreements were resolved in discussion with a third investigator. The 2 researchers used the same standardized collection form to independently extract information from each enrolled study. Data concerning study quality, population characteristics and year of publication as well as interventions and outcomes were extracted.

The quality and the risk of bias of RCTs was assessed by Cochrane Collaboration's tool.^[13]^

### 2.4 Statistical analysis

The meta-analysis was performed according to PRISMA checklist.^[14]^ For dichotomous data, treatment effects were expressed as odds ratio (OR). For continuous (mean difference) data, treatment effects were expressed as weighted mean difference (WMD). 95% Confidence interval (CI) was used for the direct meta-analysis and credible intervals (Crl) for the estimates the network meta-analyses. Heterogeneity within each pair-wise comparison when 2 or more trials were available for the comparison was accessed by Cochran *Q* test and measured by the *I*^2^ statistic. Interpretation of the *I*^2^ values was made by assigning attributes of low, moderate, and high in case of 0% to 25%, 25% to 50%, and above 75%, respectively.^[15,16]^

Firstly, we performed a traditional pair-wise meta-analysis with Stata 12 (Stata Corp, College Station, TX), synthesizing studies that compared the same reconstruction method with a random-effect model.

The network meta-analyses using the Bayesian Methods^[8]^ was performed in Stata 12 (Stata Corp), JAGS and R (version x64 3.3.3) with the gemtc package (version: 0.8–2) and rjags package (version: 4–6) with a random-effect model. The inconsistency of our results was confirmed by the node-splitting method and its Bayesian *P* value,^[17]^ comparing the direct and the indirect estimates for each comparison. *P*-value < .05 indicates a significant inconsistency. We estimated the potential ranking probability of treatments by calculating the surface under the cumulative ranking curve (SUCRA) for each intervention.^[18]^ The SUCRA index ranges between 0 and 1, the treatments with higher SUCRA values are considered to have better efficacy.

## 3 Result

### 3.1 Study selection and characteristics

**Figure 1:**
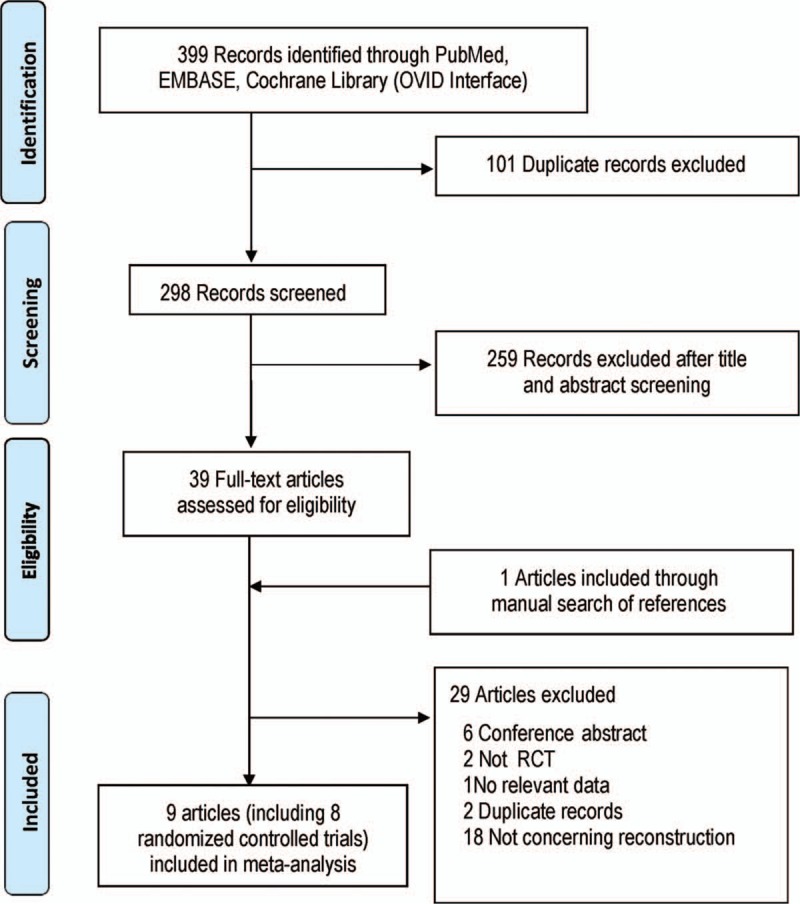
Study flow diagram.

A total of 399 articles considered to be potentially relevant were identified from various databases including PubMed, EMBASE (Ovid), and Cochrane Library (Ovid), 9 studies^[19–27]^ meeting the inclusion criteria were included in this meta-analysis. Literatures screening process is shown in Fig. 1.

**Figure 2:**
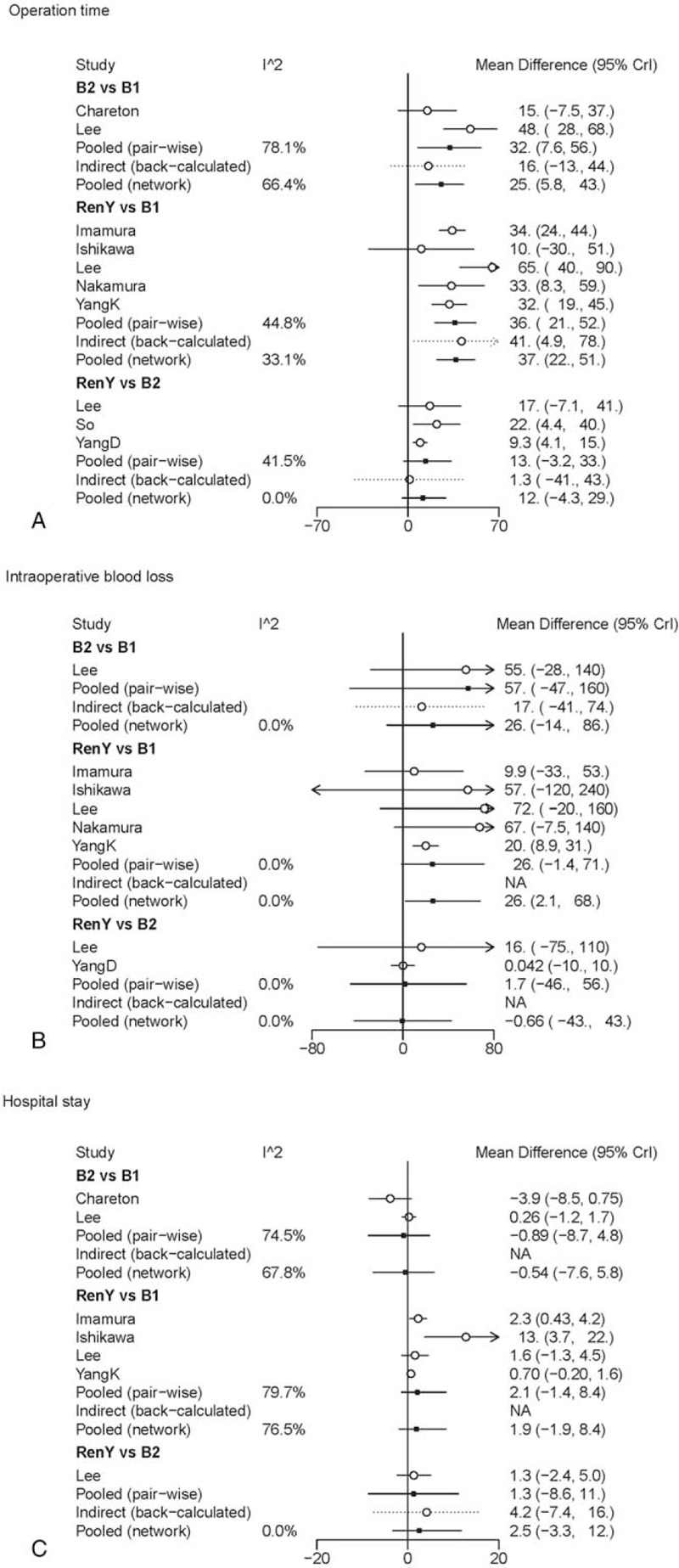
Network plot of the comparisons for the Bayesian network meta-analysis. (A) Operation time; (B) intraoperative blood loss; (C) hospital stay; (D) overall postoperative morbidity; (E) delayed gastric emptying; (F) anastomotic leakage; (G) anastomotic stricture; (H) bile reflux; (I) food residual; (J) remnant gastritis; (K) reflux esophagitis.

The characteristics of included studies are summarized in Table 1. In total, our analysis included 1161 patients: 519 treated with R-Y reconstruction; 398, surgery with B-I reconstruction; 244, surgery with B-II reconstruction. Three nodes were compared, and the network plot of all the comparisons analyzed are shown in Fig. 2. The size of the nodes and the thickness of the edges are weighted according to the number of studies evaluating each treatment and direct comparison, respectively.

**Table 1:**
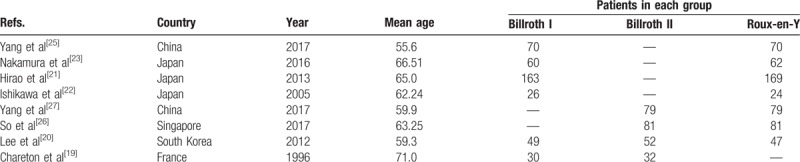
Characteristics of included studies.

### 3.2 Comparisons of surgical characteristics

**Figure 3:**
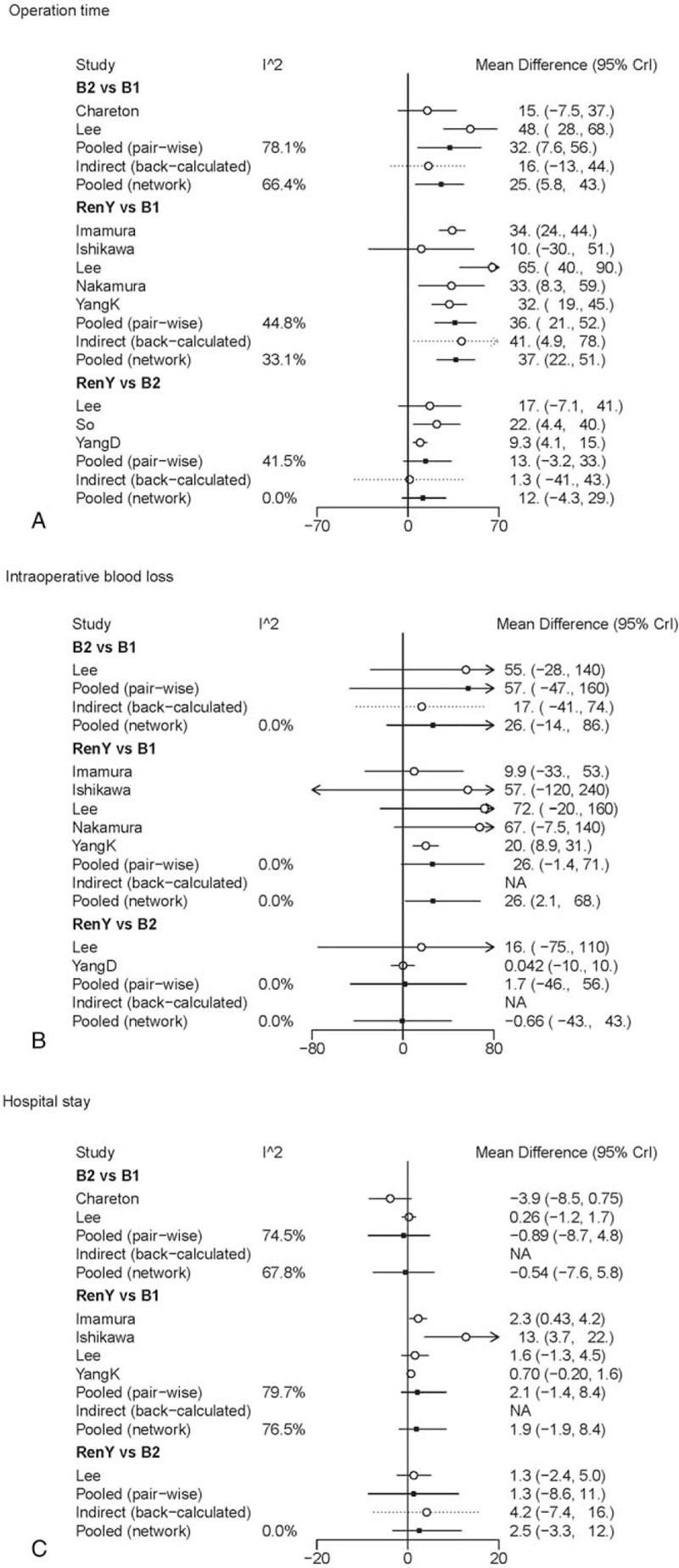
Forest plot for comparison of surgical characteristics. (A) Operation time; (B) intraoperative blood loss; (C) hospital stay.

In pair-wise meta-analysis, B-II and R-Y reconstruction exerted a trend of prolonged operation time when compared with B-I reconstruction (WMD 32, 95% CI: 7.5–56; WMD 36, 95% CI: 21 to 52), and R-Y reconstruction showed no statistical significance as compared with B-II reconstruction (WMD 13, 95% CI: −3.2 to 33). The results of comparisons of operative blood loss and hospital stay in our network meta-analysis suggested there were no significant differences among the 3 procedures. A graphical assessment of local heterogeneity and comparison between pair-wise meta-analysis and network meta-analysis for surgical characteristics is presented in Fig. 3.

The results of comparisons of surgical characteristics in our network meta-analysis are shown in Table 2. B-I reconstruction was associated with a significant reduction in operation time (WMD, −37, 95% CrI: −51 to −22) and intraoperative blood loss (WMD, −27, 95% CrI: −70 to −1.8), as compared with R-Y reconstruction. The duration of operation and operative blood loss were similar for B-II and R-Y reconstruction. No significant differences were observed between the groups regarding hospital stay, and the SUCRA values of 0.76 and 0.64 for B-II and B-I, respectively, suggested that these were the 2 procedures with the highest chance of improving hospital stay.

**Table 2:**
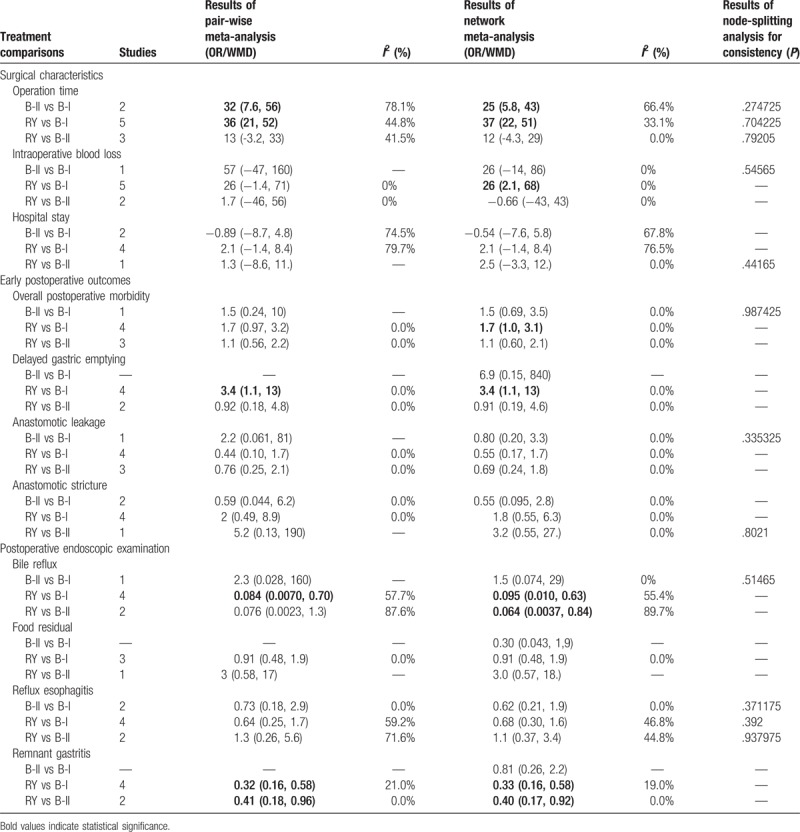
Summary of pair-wise meta-analysis and network meta-analysis results for the 3 reconstruction methods and the 10 outcomes.

B-I reconstruction seemed to be the most effective one since its SUCRA values for all the perioperative effects exceeded 0.6 (Table 3).

**Table 3:**
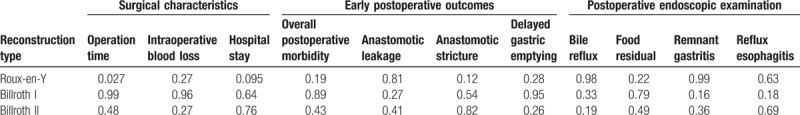
Surface under the cumulative ranking curve (SUCRA) results for all outcomes.

### 3.3 Comparisons of early postoperative outcomes

**Figure 4:**
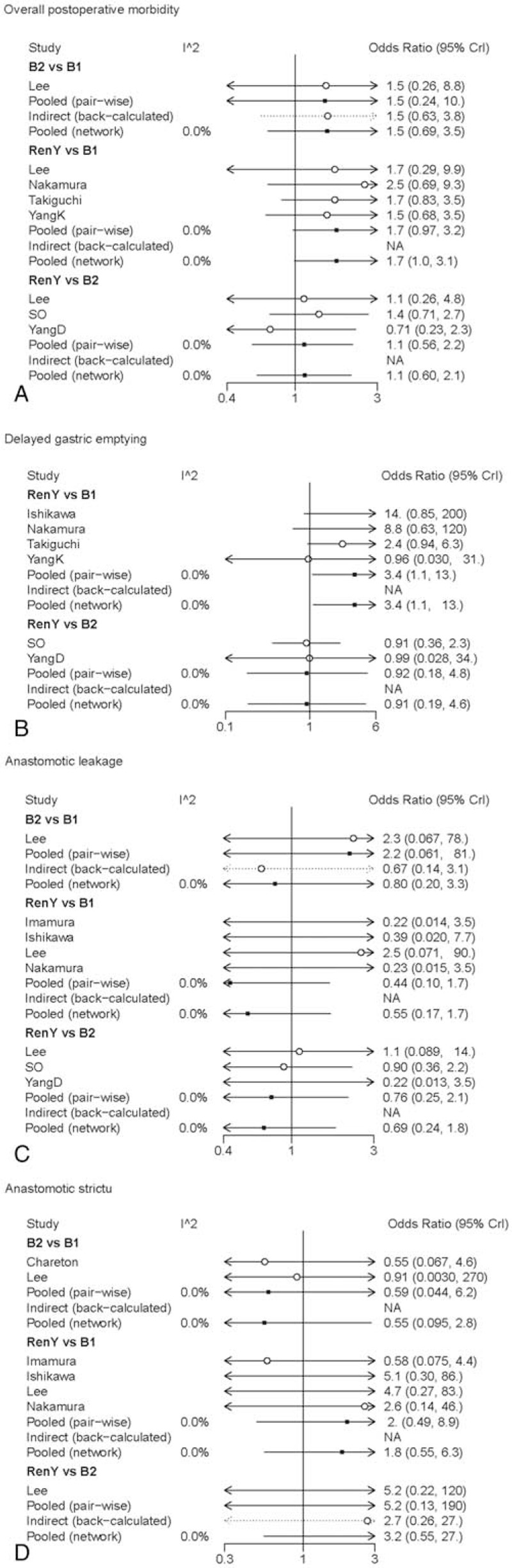
Forest plot for comparison of early postoperative outcomes. Odds ratio < 1 indicates superiority of first intervention over second intervention. (A) Overall postoperative morbidity; (B) delayed gastric emptying; (C) anastomotic leakage; (D) anastomotic stricture.

The results of pair-wise meta-analysis demonstrated that R-Y reconstruction could significantly increase the risk of delayed gastric emptying when compared with B-I reconstruction (OR 3.4, 95% CI: 1.1–13). The heterogeneity and the forest plot of comparison between pair-wise meta-analysis and network meta-analysis for early postoperative outcomes is presented in Fig. 4.

As for the results of network meta-analysis, B-I reconstruction seemed to have a trend of improved overall postoperative morbidity, however fail to get a statistical significance (OR 1.6, 95% Crl: 0.92–2.8; SUCRA = 0.89). The R-Y group presented with a higher frequency of delayed gastric emptying as compared with the B-I group (OR 3.4, 95% Crl: 1.1–13; SUCRA = 0.28). B-I reconstruction ranked the best in delayed gastric emptying (SUCRA = 0.95). No significant differences were observed among the groups in terms of anastomotic leakage and anastomotic stricture. The results of SUCRA suggested that B-II reconstruction ranked the highest in anastomotic stricture (SUCRA = 0.82).

### 3.4 Comparisons of postoperative endoscopic examination

**Figure 5:**
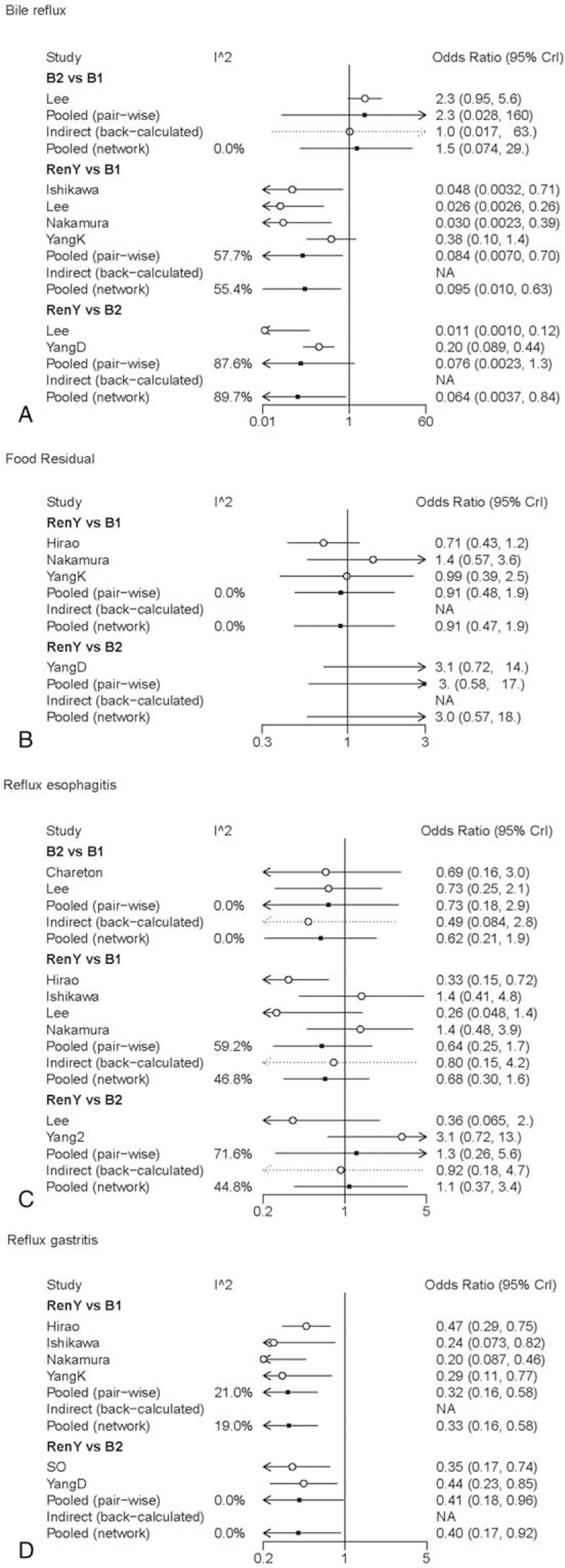
Forest plot for comparison of postoperative endoscopic examination. Odds ratio <1 indicates superiority of first intervention over second intervention. (A) Bile reflux; (B) food residual; (C) reflux esophagitis; (D) remnant gastritis.

Pair-wise meta-analysis, as shown in Table 2, indicated that R-Y reconstruction had significant superiority over B-I and B-II reconstruction in remnant gastritis (OR 0.32, 95% CI: 0.16–0.58; OR 0.41, 95% CI: 0.18–0.96, respectively). Besides, R-Y reconstruction could also significantly improve the bile reflux as compared with B-I reconstruction (OR 0.084, 95% CI: 0.0070–0.70). The graphical assessment of heterogeneity and comparison between direct meta-analysis and network meta-analysis for postoperative endoscopic examination is presented in Fig. 5.

Network meta-analysis revealed that R-Y reconstruction was superior to B-I and B-II reconstruction in terms of frequency of bile reflux (OR 0.095, 95% Crl: 0.010–0.63; SUCRA = 0.33; OR 0.064, 95% Crl: 0.0037–0.84; SUCRA = 0.19, respectively) and the incidence of remnant gastritis (OR 0.33, 95% Crl: 0.16–0.58; SUCRA = 0.33; OR 0.40, 95% Crl: 0.17–0.92; SUCRA = 0.36, respectively). No significant differences were observed among the groups in terms of reflux esophagitis and food residual (Table 2). The SUCRA value suggested that R-Y and B-II reconstruction ranked the highest in reflux esophagitis (SUCRA = 0.63 and SUCRA = 0.69, respectively).

R-Y reconstruction seemed to be the most effective one since its SUCRA values for 3 of 4 postoperative endoscopic examination exceeded 0.6.

### 3.5 Quality of evidence

**Figure 6:**
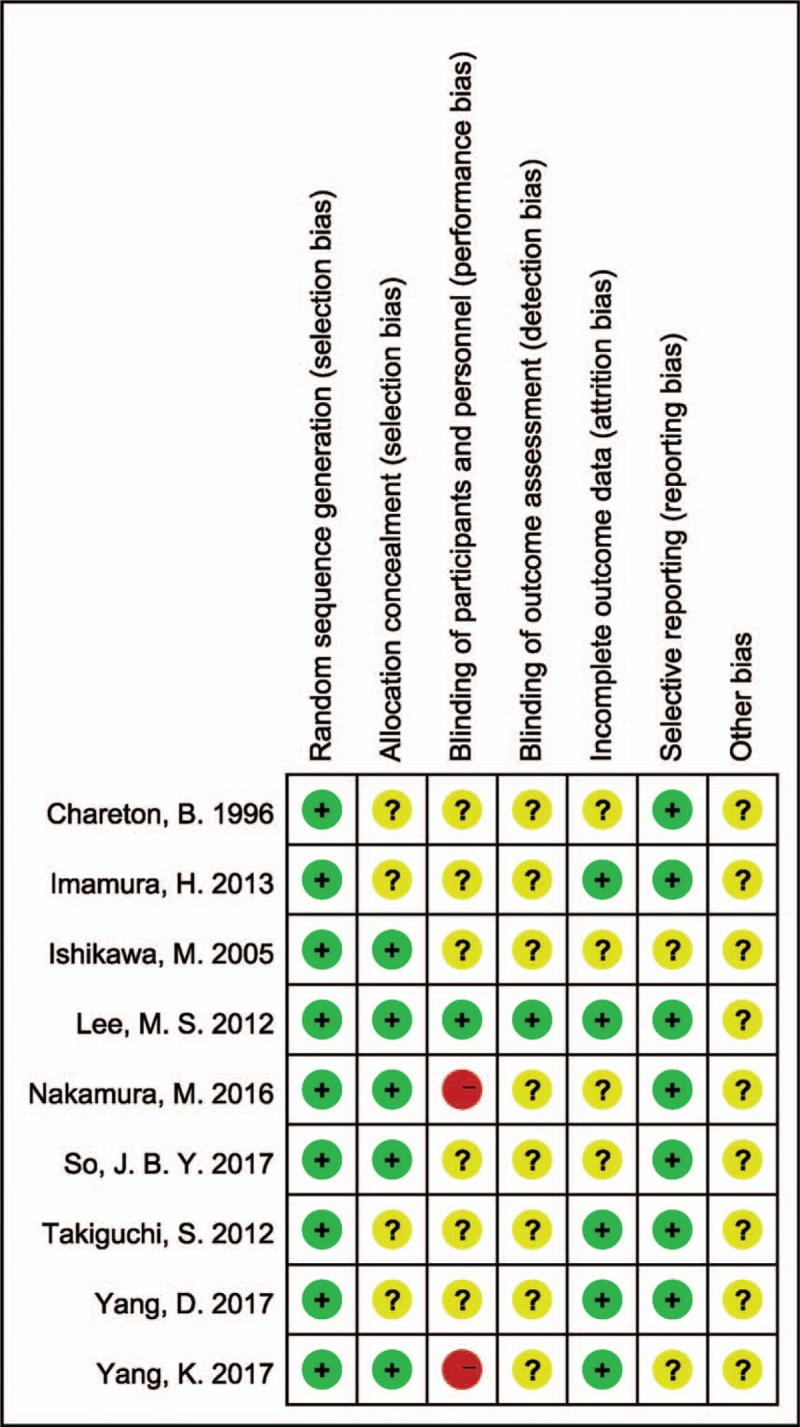
Risk of bias graph.

The bias assessment for eligible RCTs included in the network meta-analysis is shown in Fig. 6 according to the Cochrane risk-of-bias tool, showing no severe risk of bias.

We also use the node-splitting analysis with *P*-value to confirm the consistency in any closed loops of the outcomes. The results are shown in Table 2. According to the results, no consistency in any closed loop was detected with relevant *P*-value lager than .05 by the node-splitting method. No significant differences between direct and indirect estimates were found in all closed loops.

## 4 Discussion

Until recently, gastrointestinal reconstruction procedure options after distal or subtotal gastrectomy for patients with GC are still controversial. B-I reconstruction has been widely performed after distal gastrectomy in Japan and Korea for the reason that most GC diagnosed in these countries are usually early-stage and its physiological advantage of maintaining a normal passage for food to pass through the duodenum.^[28]^ However, patients undergoing B-I and B-II reconstruction frequently suffer from the reflux symptoms. On the contrary, the R-Y reconstruction is reported to be superior to the conventional B-I and B-II reconstruction in preventing reflux symptoms and in preventing impeding gastritis^[29]^ which increase the risk of carcinogenesis at the gastric remnant.^[30]^ However, it is more complicated to perform with more procedures.

In this systematic review and network meta-analysis, we focused on perioperative effects and postoperative effects of the 3 reconstruction methods for patients with GC.

In our analysis of surgical characteristics, B-I reconstruction was associated with a significant reduction in operation time as compared with B-II and R-Y reconstruction, and B-I reconstruction reduced the operation blood loss as compared with R-Y. However, whether blood transfusions affect the survival of GC patients is still controversial.^[31,32]^

For the results of early postoperative outcomes, the Billroth I reconstruction which allowed food to pass through the duodenum was superior to the R-Y procedure in terms of delayed gastric emptying, confirming the physiological advantage of B-I reconstruction. Several studies reported that part of patients suffer the R-Y stasis syndrome with functional obstruction of the Roux limb, which is caused by separation of the Roux limb from the natural small-bowel pacemaker.^[6,33,34]^

When we were focusing on the patients’ postoperative endoscopic examination, R-Y anastomosis was superior to B-I and B-II in terms of frequency of bile reflux and remnant gastritis, which was consistent with previous reports.^[35]^ Previous study also suggested that the method could also improve quality of life and reduce the risk of carcinogenesis in the gastric remnant.^[36]^ However, there was no statistical difference in reflux esophagitis and food residual among the 3 groups.

Nonetheless, some limitations in the present work merit further discussion. First, all networks had only one closed loops of evidence formed by different independent trials in these networks. Second, the perioperative and postoperative effect must be balanced against survival benefits. However, we could not analyze the survival benefits of the relevant reconstruction types on account of the shortage of the data of survival of some studies enrolled in this meta-analysis.

Despite these limitations, there are several strengths of our study. The systematic review and network meta-analysis incorporates all currently available RCTs concerning different types of reconstruction methods and to the best of our knowledge, and this is the first attempt to systematically and quantitatively review the literature in this field. Moreover, inclusion criteria for enrolled trials were very similar, producing homogeneous populations and study characteristics for our study. No inconsistent results were observed in the calculation, which strengthened the validity of our results.

In conclusion, R-Y reconstruction is superior to B-I and B-II reconstruction in terms of preventing bile reflux and remnant gastritis, B-I and B-II anastomosis could be considered as the substitute in consideration of technical simplicity. As for postoperative morbidity and the advantage of physiological food passage, B-I method is the choice.

## Author contributions

Bo Zhang, Zhaolun Cai, Ye Zhou designed the study. Chenxiao Wang and Xiaonan Yin screened studies and extracted data. Disagreements were resolved by discussion with Yuan Yin. Zhaolun Cai did the statistical analyses and prepared figures. Yiqiong Yin, Chenxiao Wang, Zhixin Chen, Yuan Yin, Chaoyong Shen, Zhaolun Cai reviewed the results, interpreted data, and wrote the manuscript. All authors saw and approved the final version of the paper.
